# Endoplasmic reticulum stress-mediated cell death in liver injury

**DOI:** 10.1038/s41419-022-05444-x

**Published:** 2022-12-19

**Authors:** Jian Zhang, Jiafu Guo, Nannan Yang, Yan Huang, Tingting Hu, Chaolong Rao

**Affiliations:** 1 School of Public Health, Chengdu University of Traditional Chinese Medicine, Chengdu, Sichuan 611137 China; 2 R&D Center for Efficiency, Safety and Application in Chinese Materia Medica with Medical and Edible Values, School of Public Health, Chengdu University of Traditional Chinese Medicine, Chengdu, Sichuan 611137 China; 3 State Key Laboratory of Southwestern Chinese Medicine Resources, Chengdu University of Traditional Chinese Medicine, Chengdu, Sichuan 611137 China

**Keywords:** Cell death, Endoplasmic reticulum

## Abstract

The endoplasmic reticulum is an important intracellular organelle that plays an important role in maintaining cellular homeostasis. Endoplasmic reticulum stress (ERS) and unfolded protein response (UPR) are induced when the body is exposed to adverse external stimuli. It has been established that ERS can induce different cell death modes, including autophagy, apoptosis, ferroptosis, and pyroptosis, through three major transmembrane receptors on the ER membrane, including inositol requirement enzyme 1α, protein kinase-like endoplasmic reticulum kinase and activating transcription factor 6. These different modes of cell death play an important role in the occurrence and development of various diseases, such as neurodegenerative diseases, inflammation, metabolic diseases, and liver injury. As the largest metabolic organ, the liver is rich in enzymes, carries out different functions such as metabolism and secretion, and is the body’s main site of protein synthesis. Accordingly, a well-developed endoplasmic reticulum system is present in hepatocytes to help the liver perform its physiological functions. Current evidence suggests that ERS is closely related to different stages of liver injury, and the death of hepatocytes caused by ERS may be key in liver injury. In addition, an increasing body of evidence suggests that modulating ERS has great potential for treating the liver injury. This article provided a comprehensive overview of the relationship between ERS and four types of cell death. Moreover, we discussed the mechanism of ERS and UPR in different liver injuries and their potential therapeutic strategies.

## Facts

- ERS mediates chain reactions mainly through three pathways of unfolded protein response.
- ERS can induce different modes of cell death, including autophagy, apoptosis, ferroptosis, and pyroptosis.
- ERS plays an important role in liver injury and its treatment and has huge prospects for application in precision medicine.

## Open questions

- How do the three signaling pathways downstream of UPR interact with each other?
- How does ERS determine the ultimate mode of cell death?
- Is the role of ERS consistent in different stages of liver injury?

## Introduction

The endoplasmic reticulum (ER) is an important continuous membranous organelle consisting of a series of flattened sacs connected to the nuclear membrane and has multiple biological functions [1], involving cholesterol and phospholipid synthesis in the lumen of the ER for the production of cell membranes [2]. It is involved in storing and releasing Ca^2+^ in the ER lumen [3], and coordinates protein processing, folding, and transport in cells [4]. Given that correctly folded proteins in cells are the basis of life activities, ER plays a crucial role in protein quality control [5]. Importantly, conditions such as genetic mutation, hypoxia, nutrient deficiency, and oxidative stress induce the occurrence of endoplasmic reticulum stress (ERS), resulting in the accumulation of unfolded and misfolded proteins in the ER lumen, thereby activating the unfolded protein response (UPR) to resist the unfavorable external environment [6]. When cells suffer ERS, the activated UPR causes two opposite outcomes. On the one hand, cells that experience short-term ERS exhibit enhanced ER ability to process proteins through the UPR to restore ER homeostasis to a physiological state, allowing cells to survive. On the other hand, cells experience severe ERS; it usually causes cell death through multiple pathways and severely damages the body [7]. There is rich literature available suggesting that ERS is closely associated with many diseases, especially during liver injury, as well as the development of neurodegenerative diseases, inflammation, and metabolic syndrome [8].

The liver is the largest metabolic organ and is rich in enzyme systems, carrying functions such as metabolism and secretion. Moreover, it is the main site of protein synthesis in the body. Therefore, a well-developed ER system is present in hepatocytes to help the liver perform its physiological functions [9]. When adverse factors stimulate the liver, it causes liver synthesis and metabolism dysfunction, triggering ERS and then activating a series of cascade reactions, causing different degrees of damage to the liver and seriously threatening human health [10]. In this article, we provide a comprehensive overview of the main signaling pathways and mechanisms of UPR induced by ERS, summarize the pathways by which ERS leads to different modes of cell death and elaborate on the role of ERS in liver injury to provide novel insights for liver injury prevention and treatment.

## Regulation of ERS and UPR

When external adverse factors stimulate the body, ERS may be caused, which may be attributed to the accumulation of unfolded and misfolded proteins in the lumen of the ER due to improper folding and processing of proteins [11]. After induction of ERS, the host usually develops a response to this stress state to restore its normal physiological function, called UPR. The UPR allows the body to maintain ER protein homeostasis through negative feedback regulation to further cope with ERS via the following mechanisms: [12] (a) Reduction of protein synthesis, degradation of most mRNA by regulated IRE1α-dependent decay (RIDD) or acting on ribosomes to prevent the translation of second messengers; (b) Preventing post-translational polypeptide chains that need to be processed and folded from entering the ER lumen to relieve the load on the ER to cope with unfolded proteins; (c) Enhancing the ability of ER to process unfolded proteins by enhancing the expression of related molecular chaperone glucose-regulated protein 78 (GRP78); (d) Transport of excessive proteins in the ER that cannot be effectively processed to the cytoplasm for degradation through relevant pathways to reduce the ER load [13]. The scavenging of proteins is mainly achieved through endoplasmic reticulum-associated degradation (ERAD), which displaces abnormally accumulated proteins from the ER lumen into the cytoplasm, and is subsequently degraded by 26s proteasome ubiquitination [4].

**Fig. 1:**
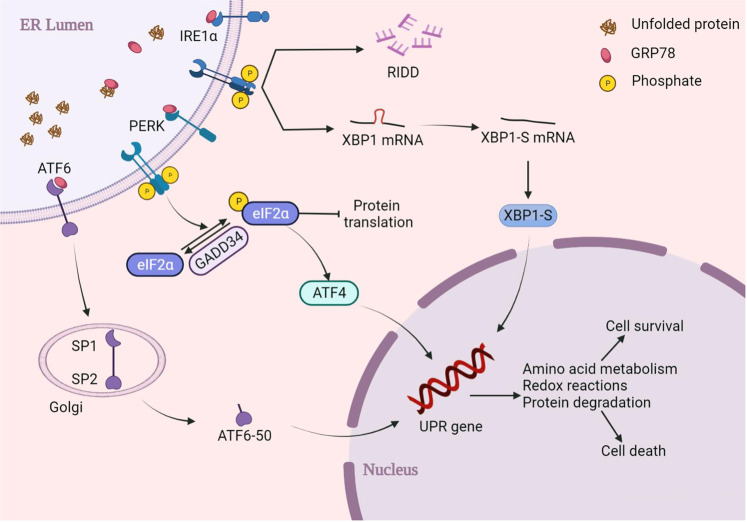
Major pathway of UPR in mammals. Three transmembrane receptors, inositol-requiring enzyme 1α (IRE1α), pancreatic endoplasmic reticulum kinase (PERK), and activating transcription factor 6 (ATF6), mediate endoplasmic reticulum stress (ERS) and unfolded protein response (UPR). It can act on the corresponding genes in the nucleus after activation through three pathways, triggering amino acid metabolism, redox reaction, and protein degradation.

Three transmembrane receptors can activate the UPR on the ER, including inositol-requiring enzyme 1α (IRE1α), pancreatic endoplasmic reticulum kinase (PERK), and activating transcription factor 6 (ATF6), and sense stress in the ER lumen to activate relevant downstream signal transduction pathways and improve the processing capacity of unfolded and misfolded proteins in the ER [14]. Currently, two theories have been postulated about how the three transmembrane receptors sense ERS. Firstly, three transmembrane receptors directly sense unfolded proteins in the ER lumen. After the unfolded proteins bind to the transmembrane receptors, oligomerization and autophosphorylation are activated, and the relevant downstream signals are triggered. Besides, under normal conditions, the ER transmembrane protein receptor binds to GRP78, a member of the heat shock proteins 70 (HSP70) family in the ER lumen, which induces inhibition of the signal receptor. When ERS occurs, GRP78 dissociates from transmembrane receptors, binds to and processes unfolded proteins, while transmembrane receptors dissociated from GRP78 undergo oligomerization and autophosphorylation, activating relevant downstream signals [15]. Meanwhile, studies have found that the IREIα transmembrane receptor in yeast is activated by direct binding to unfolded proteins rather than by dissociation of GRP78 [16]. However, the activation of IREIα and PERK in mammals appears to be primarily triggered by the dissociation of GRP78 (Fig. 1) [17].

### IRE1α signaling pathway

IRE1α is the most evolutionarily conserved ER stress sensor and is a type I transmembrane protein in the ER of all eukaryotes, including yeast and humans [18]. The N-terminal ER lumen domain is a peptide-binding domain similar to the antigen-presenting major histocompatibility complex, which can sense unfolded and misfolded proteins in the ER. In the cytoplasm, it has two domains, the serine/threonine protein kinase domain and the RNase domain. When ERS occurs, IRE1α undergoes oligomerization and trans-autophosphorylation after dissociation from GRP78 in the ER lumen, leading to activation of endoribonuclease and cleavage of its target gene X-box-binding protein 1 (XBP1) mRNA. Delivery of cleaved XBP1 into the nucleus caused upregulation of target genes associated with unfolded UPR. In addition, IRE1α activation can cause the degradation of mRNAs and further reduce the protein load, a process known as Regulated IRE1α-dependent decay **(**RIDD) [19].

### PERK signaling pathway

PERK is also a type I transmembrane protein on the endoplasmic reticulum membrane, and its domain shares high structural similarity with IRE1α [20]. During ERS, PERK undergoes dissociation from GRP78, oligomerization, and trans-autophosphorylation activates its kinase domain and phosphorylates its major target substrate eIF2α, terminating the translation of most mRNAs. However, it selectively enhances the translation of activating transcription factor 4 (ATF4) and promotes the expression of antioxidant responses, amino acid biosynthesis, and transport genes to maintain cell survival [21]. In addition, ATF4 promotes the expression of growth arrest and DNA damage inducible-protein 34 (GADD34), which interacts with protein phosphatase 1 (PP1) and then dephosphorylates p-eIF2α to alleviate endoplasmic reticulum stress [22].

### ATF6 signaling pathway

ATF6 is a type II transmembrane protein with a large domain in the ER that acts as a transcription factor. In an unstressed state, binding to GRP78 is inhibited. During ERS, ATF6 dissociates from GRP78, which participates in the processing and folding of unfolded proteins, and ATF6 is transported to the Golgi body through vesicles. Current evidence suggests that ATF6 is cleaved by serine site-1 protease (S1P) and metalloprotease site-2 protease (S2P) in the Golgi to generate an active 50 kDa free N-terminal fragment, which is transported to the nucleus, binds to genes related to the ERS response element and regulates their transcription and expression [23].

## Cell death mediated by ERS and UPR

### Autophagy

Autophagy is an evolutionarily highly conserved process in eukaryotes that is used to degrade and recycle intracellular biological macromolecules and damaged organelles, which is of great significance for maintaining cellular homeostasis [24]. There are three types of autophagy: macroautophagy, microautophagy, and chaperone-mediated autophagy. ERS is often associated with non-selective macroautophagy. Current evidence suggests that both ERS and autophagy are protective mechanisms that involve the degradation of intracellular substances to maintain the normal physiological functions of cells. Therefore, autophagy can be regarded as another approach to removing excess proteins from the ER besides the ERAD process.

**Fig. 2:**
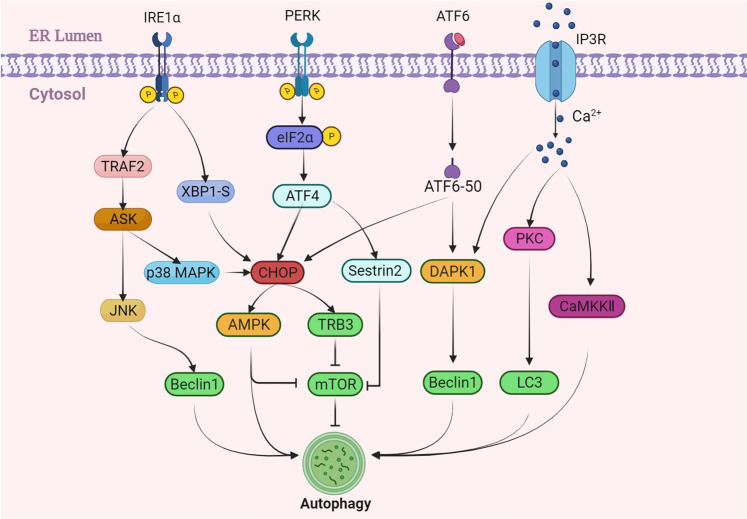
The UPR activates autophagy. ERS mainly mediates autophagy through four pathways: PERK, IRE1α, ATF6, and Ca^2+^, of which the key regulator is C/EBP homologous protein (CHOP). When ERS is activated, IRE1α acts on Beclin1 through the tumor necrosis factor receptor-associated factor 2 (TRAF2) and Jun N-terminal kinase (JNK) pathway to promote autophagy; ATF6 activates its downstream death-associated protein kinase 1 to activate autophagy through Beclin1; all three UPR pathways can regulate autophagy by activating CHOP protein. In addition, Ca^2+^ can also participate in autophagy through protein kinase C (PKC) and calcium/calmodulin-dependent protein kinase II (CaMKII).

During the process of ERS-induced autophagy, the three branches of UPR regulate the occurrence of autophagy through corresponding pathways. After the UPR pathway was activated, C/EBP homologous protein (CHOP) activates its downstream AMP-activated protein kinase (AMPK) or tribbles homologue3 (TRB3) to inhibit the mammalian target of rapamycin complex 1 (mTORC1), promoting the formation of unc-51-like kinase 1 (ULK1) complex and activate autophagy [25]. IRE1α pathway can activate tumor necrosis factor receptor-associated factor 2 (TRAF2) and then activates apoptosis signal-regulating kinase (ASK), leading to the activation of c-Jun N-terminal kinase (c-JNK), which mediate the phosphorylation of Bcl2, and disrupt the interaction between Bcl2 and Beclin1, leading to the release of Beclin1, which enhances basal autophagy [26]. An increasing body of evidence suggests that the IRE1α-TRAF2 pathway can also promote the extension and expansion of the autophagosome membrane by promoting the formation of the ATG complex and LC3. In addition, the PERK-eIF2α-ATF4 pathway of UPR leads to the expression of Sestrin2 and DDIT4-related genes, inhibits mTORC1 activity, and induces autophagy (Fig. 2) [27]. The release of Ca^2+^ in the ER can also activate corresponding factors such as death-associated protein kinase 1 (DAPK1), protein kinase C (PKC), and Calcium/calmodulin-dependent protein kinase II (CaMKII) to participate in the regulation of autophagy [28–30].

Interestingly, growing evidence suggests that ERS-induced autophagy is extensively involved in apoptosis. Hai et al. found that 3-acetyldeoxynivalenol could induce PERK pathway activation, which leads to autophagy and apoptosis of mouse hepatocytes, thereby causing liver injury. Interestingly, autophagy inhibitors reduce cell apoptosis caused by 3-acetyldeoxynivalenol, indicating that ERS-induced autophagy is closely related to cell death [31].

### Apoptosis

**Fig. 3:**
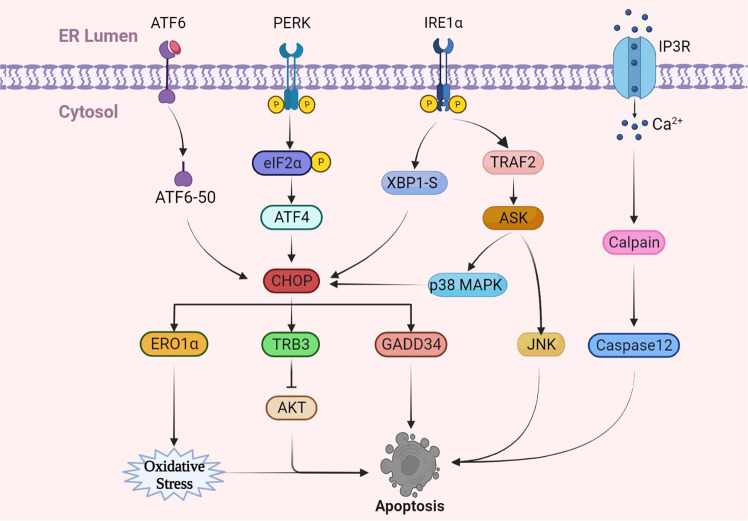
The UPR induces activation of apoptosis. Apoptosis induced by ERS is similar to autophagy and is mediated by four major pathways, PERK, IRE1α, ATF6, and Ca^2+^. Among them, the CHOP pathway is the main factor of apoptosis, and all three transmembrane sensors can induce apoptosis. ERS can also induce apoptosis through the IRE1α-TRAF2-JNK pathway. In addition, apoptosis triggered by the Ca^2+^ pathway is induced by the activation of its specific factor, caspase12.

Apoptosis is programmed cell death characterized by DNA degradation, nucleus concentration and fragmentation, and the formation of apoptotic bodies, with an intact cell membrane but no secondary inflammatory reactions. Apoptosis is an important process to maintain life activities mainly caused by two pathways, one caused by the extracellular death ligand-receptor pathway and the other caused by the intracellular pathway. ERS represents an important way to induce apoptosis in cells. When ERS occurs in cells, three transmembrane protein receptors on the ER membrane are activated, thus causing apoptosis (Fig. 3).

When ERS occurs, IRE1α is activated, interacting with CHOP through XBP1 and activating the downstream c-JNK or p38 mitogen-activated protein kinases (p38-MAPK) gene expression through TRAF2-ASK pathway, thus leading to apoptosis [32–34]. PERK is activated by dimerization and trans-autophosphorylation, which phosphorylates eIF2α and inhibits the translation of most proteins. However, this inhibition of translation is not complete, and mRNAs carrying specific regulatory sequences in the 5′ untranslated regions can escape the inhibition of translation mediated by phosphorylated eIF2α and continue synthesizing ATF4. After ATF4 enters the nucleus, it can upregulate the expression of CHOP and the downstream factor GADD34, thereby changing the expression of Bcl2 apoptosis family-related proteins and causing apoptosis. Besides, GADD34 can bind to the α subunit of PP1 to dephosphorylate phosphorylated eIF2α and continue the protein translation process, which can aggravate ERS and promote cell apoptosis [35]. In addition, CHOP-induced apoptosis is associated with endoplasmic reticulum oxidoreductase 1α (ERO1α), which can increase the expression of the ERO1α (ER reductase) gene (functions as an oxidative enzyme of protein disulfide isomerase (PDI)), resulting in a large amount of H_2_O_2_ in the ER [36]. The hyperoxidized state of the ER leads to the leakage of H_2_O_2_ into the cytoplasm, inducing oxidative stress and a series of apoptotic and inflammatory responses [37]. After ERO1α activates inositol 1,4,5-trisphosphate receptor 1 (IP3R1), it opens the Ca^2+^ channel on the ER membrane, releases Ca^2+^ into the cytoplasm, then activates the CaMKII and NADPH oxidase on the cell membrane, which can both promote further production of ROS and induce apoptosis [38]. CHOP-mediated TRB3-PKB and other pathways induce apoptosis, but its specific mechanism remains largely unclear [7].

Caspase12 is a key specific molecule located on the ER membrane that regulates apoptosis and is activated by the CHOP-ERO1α pathway [39]. After the CHOP-ERO1α pathway releases Ca^2+^ into the cytoplasm to activate calpain, caspase12 is cleaved by calpain to activate it. The activated caspase12 is translocated from the ER membrane to the cytoplasm, activating other caspase effector proteins and causing apoptosis [40, 41].

### Ferroptosis

Ferroptosis is a cell death mode different from apoptosis and autophagy. Ferroptosis is characterized by the action of ferrous iron or lipoxygenase, which catalyzes the high expression of unsaturated fatty acids on the cell membrane to undergo lipid peroxidation and a decrease in glutathione peroxidase 4 (GPX4), the regulatory core enzyme of the antioxidant system, thereby inducing cell death [42]. Elevation of intracellular Fe^2+^ is the main cause of ferroptosis [43], as well as nuclear factor E2-related factor 2 (Nrf2), which is involved in regulating cellular iron metabolism, and plays an important role in the mechanism of ferroptosis. Mounting studies have found that ERS can cause Fe^2+^ accumulation and lipid peroxidation through the PERK-Nrf2-HO-1 pathway, thereby causing ferroptosis [43, 44].

**Fig. 4:**
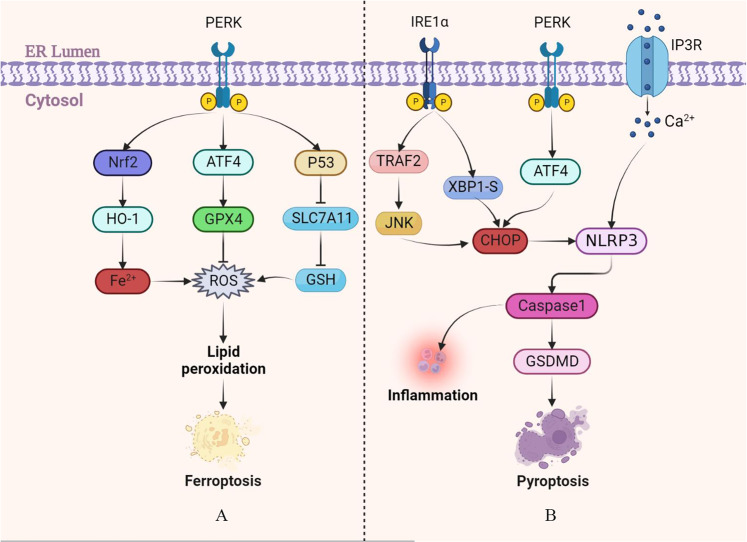
Ferroptosis and pyroptosis involved in the UPR pathway. **A** The ferroptosis pathway caused by ERS is mainly induced by the PERK pathway, which involves the metabolism of Fe^2+^ in cells, the accumulation of lipid peroxides caused by glutathione depletion, and eventually leads to the occurrence of ferroptosis. **B** Pyroptosis is mainly mediated by PERK, IRE1α, and Ca^2+^ pathways of ERS. After PERK and IRE1α are activated, the downstream NOD-like receptor thermal protein domain associated protein 3 (NLRP3) is activated to induce inflammation and pyroptosis. In addition, Ca^2+^ can also induce pyroptosis by directly acting on NLRP3.

In addition, polyunsaturated fatty acids (PUFAs) are major substrates of lipid metabolism pathways involved in the ferroptosis mechanism. Due to the presence of multiple unsaturated bonds, PUFAs are prone to lipid peroxidation, leading to the accumulation of lipid free radicals, which is key in causing ferroptosis. Under the action of Acyl-CoA synthetase long-chain family member 4 and lysophosphatidylcholine acyltransferase 3, the key enzymes in the synthesis and remodeling of phospholipid ethanolamine (PEs), PUFAs are esterified to form PEs, which are then oxidized to lipid peroxidation products by lipoxygenases or CYP450 oxidoreductase; when these peroxides accumulate, ferroptosis occurs [45]. The amino acid metabolic pathway in ferroptosis is mainly involved by GPX4 and glutathione (GSH), both of which are involved in the metabolism of peroxides in the body. When in vivo GPX4 activity is too low or GSH synthesis is blocked, lipid peroxides accumulate, causing ferroptosis [46]. Cysteine/glutamate transporter (System Xc^-^) is a heterodimer composed of heavy chain solute carrier family 2 member 2 (SLC2A2) and light chain solute carrier family 7 member 11 (SLC7A11) that exists on the cell surface and participates in the synthesis of intracellular GSH to induce ferroptosis [47]. Similarly, in the PERK-ATF4 pathway of ERS, ATF4 activation enables HSP5A to combine with GPX4 to form a complex, inhibiting the degradation of GPX4 and ferroptosis. PERK pathway can also inhibit the generation of System Xc^-^ through the p53 gene to reduce the synthesis of GSH and ultimately promote ferroptosis [48]. In addition, ATF4 can increase the expression level of System Xc^-^ through transcriptional regulation and inhibit the occurrence of ferroptosis [46]. In recent years, research on ERS and ferroptosis has mainly focused on the PERK pathway, suggesting differences in the cell death patterns mediated by the three UPR pathways (Fig. 4A).

### Pyroptosis

Pyroptosis refers to the pro-inflammatory necrosis of cells based on the pore-forming activity of the Gasdermin protein family. Its main characteristics are nuclear condensation, random fragmentation and degradation of chromatin DNA, and the formation of numerous pores in the cell membrane, which stimulate the inflammatory response, resulting in cell swelling and rupture [49]. The main mechanism of pyroptosis involves the recruitment of inactive pro-caspase1, which is cleaved into active caspase1 by the multi-protein complex inflammasome after sensing external stimuli. Caspase1 can cleave and activate GSDMD protein to N-GSDMDS, which can bind to the cell membrane, punch 1–2-nm-sized holes, break the integrity of the cell membrane, lead to the outflow of cell contents, causing osmotic pressure changes and eventually resulting in cell swelling and rupture [50]. At the same time, caspase1 cleaves inactive pro-IL-1β and pro-IL-18 to produce active inflammatory factors IL-1β and IL-18 and further propagate the inflammatory response [51].

Current evidence suggests that IRE1α can activate the thioredoxin interacting protein (TXINP)-NOD-like receptor thermal protein domain associated protein 3 (NLRP3) pathway, promote the release of GSDMD protein, and aggravate the occurrence of pyroptosis [52, 53]. In addition, it has been reported that ERS can promote the expression of CHOP through the PERK and eIF2α pathways rather than the ATF6 pathway, and the increased expression of CHOP can promote the production of NLRP3, thereby inducing pyroptosis [54]. Another study reported that excessive intake of Cu^2+^ can cause ER expansion, increase the expression of GRP78 and caspase1 proteins, and increase the expression of ERS and pyroptosis-related proteins and mRNAs, indicating that Cu^2+^ can cause pyroptosis through ERS. In addition, they found that both 4-Phenylbutyric acid (4-PBA, ERS inhibitor) and MKC-394 (IRE1α inhibitor) could inhibit ERS to alleviate the occurrence of pyroptosis [55]. These results suggest that ERS regulates copper ion-induced pyroptosis through the IRE1α-XBP1 pathway (Fig. 4B). Interestingly, copper-mediated cell death (cuproptosis) has become a research hotspot in recent years. Tsvetkov et al. proposed that excessive intracellular Cu^2+^ could cause the formation of disulfide bonds and aggregation of lipoacylated proteins in TCA, elevating HSP70 to cause cuproptosis [56]. Although the association between ERS and cuproptosis remains unclear, the landmark protein GRP78 in ERS is indeed a member of the HSP70 family, and ERO1α during ERS can catalyze the formation of protein disulfide bonds to promote protein folding. Therefore, it is speculated that ERS may play a certain role in the new cell death mode-cuproptosis [57].

When the body is stimulated by external adverse conditions and ERS occurs, UPR is activated to restore the body to its normal physiological state. However, when ERS is too strong or lasts too long beyond the adaptive regulation range of ER, it can lead to severe imbalances of ER homeostasis, leading to cell death. At present, research on ERS-mediated cell death has mainly focused on autophagy and apoptosis, yet ferroptosis and pyroptosis are also involved.

Autophagy can play a regulatory role in maintaining cell homeostasis. Studies have found that the administration of exogenous chemicals to hepatocytes can inhibit the occurrence of autophagy and then induce cell death, which can be inhibited by rapamycin (autophagy inducer) [58]. It is widely thought that a mutual regulatory relationship exists between ERS-induced autophagy and apoptosis. Overwhelming evidence substantiates that ERS-induced autophagy is generally inhibited by exogenous chemical damage, including autophagy initiation, elongation, and autophagy-lysosome fusion, which enhances apoptosis [31, 59, 60]. However, autophagy and apoptosis caused by ERS mediated by cantharidin can jointly promote cell death and induce nephrotoxicity, which may be due to the fact that CHOP, a key factor downstream of ERS, can prevent the binding of Bcl2 and Beclin1, release Beclin1 and inhibit the expression of Bcl2, thus promoting autophagy and apoptosis [61]. In addition, it has been found that ERS can regulate ATF4 through the PERK pathway to promote autophagy and inhibit cell death in hepatocellular carcinoma cells. On the other hand, it can regulate changes in p53 through the PERK pathway and affect the occurrence of apoptosis and ferroptosis [48]. This phenomenon also revealed the regulatory role of ERS in different cell death modes. However, the specific mechanisms of different cell death modes induced by ERS warrant further exploration, especially in ferroptosis, pyroptosis, and even cuproptosis.

## The important role of endoplasmic reticulum stress in liver injury

### Transcription factors/genes defect and hepatic steatosis

Fatty liver disease caused by lipid metabolism disorder is closely related to alcohol, a high-fat diet (HFD), obesity, and drug therapy [62]. A growing literature suggests that ERS-induced UPR plays an important role in lipid metabolism homeostasis. It has been found that the deletion of transcription factors (TFs) or genes can induce or aggravate ERS and cause non-alcoholic fatty liver disease (NAFLD). Although the sources of these TFs are different, after knockout, they can affect lipid metabolism through the ERS pathway, resulting in lipid accumulation and hepatic steatosis (Table 1). Liver development and function are regulated by many TFs, such as forkhead box protein A2 (FOXA2), forkhead box protein A3 (FOXA3), hepatocyte nuclear factor 1 homeobox α (HNF1α), and farnesoid X receptor (FXR). HNF1α is a transcription factor abundant in the liver and essential for glucose metabolism, detoxification, and plasma protein synthesis [63]. Studies have found that HNF1α can increase GRP78 to alleviate ERS and inhibit hepatocyte apoptosis. When HNF1α is knocked down, it continues to induce ERS, inflammation, and hepatocyte death, leading to liver injury [64]. Another study found that FXR is closely related to ERS-induced inflammation and liver injury. FXR knockdown exacerbated the occurrence of ERS, leading to the activation of NLRP3 and TXNIP, which in turn activated the inflammatory response and caused hepatocyte death [65]. These experiments indicated that transcription factors HNF1α and FXR in hepatocytes could interact to alleviate the occurrence of liver injury. It is widely thought that ERS induced by aging and a high-fat diet can reduce the expression of HNF1α through its downstream activation of c-JNK and p38-MAPK and then inhibit the expression of FXR, increase triglyceride production, promote lipid deposition, and cause hepatic adipose disease [66, 67]. These studies further suggest their role in protecting the liver. FOXA2 is an important transcription factor essential in the growth and development of liver. Studies reported that ERS induced by FOXA2 knockdown in hepatocytes could cause cholestasis and bile acid toxicity, increase lipid accumulation in hepatocytes and cause hepatic steatosis [68, 69]. Interestingly, it has been documented that FOXA3 exerts opposite effects to FOXA2. FOXA3 expression is usually regulated by XBP1. When FOXA3 expression is increased, sterol regulatory element binding protein 1c (SREBP1c) expression is also increased, leading to lipid accumulation and hepatic steatosis, alleviated after FOXA3 knockout [70]. In addition to the above TFs, the knockout of TFs can also cause lipid deposition and hepatic steatosis by affecting ERS and regulating the activities of enzymes related to lipid metabolism. For example, XBP1, as a regulator of ERS, can alleviate ERS and prevent hepatic steatosis, while in XBP1-knockout mice, long-term unrelieved ERS can promote hepatic steatosis [71]. Therefore, the above experiments confirmed that ERS is mainly involved in hepatic steatosis caused by TFs deficiency, suggesting it represents a potential means to treat fatty liver disease by regulating the expression of UPR and its downstream factors.

**Table 1:** TFs deficiency induced ERS causing dysregulated hepatic steatosis.

Incentive^*^	TF/Gene	Cell/Animal	Consequences	References
LPS/CCl_4_	HNF1α (−)	LO2 cells	Exacerbating ERS, inducing hepatocyte apoptosis and aggravating liver injury.	[64]
HFD/Aging/TM	FXR (−)	Fish and C57BL/6 mice	Exacerbating ERS, inducing inflammation, leading to hepatic steatosis and liver injury.	[65–67]
KO	FOXA2 (−)	iPSC-derived hepatocytes and Cre mice	Activating ERS, causing hepatic steatosis, bile acid toxicity.	[68, 69]
HFD/TM	FOXA3 (−)	Mice	Alleviating ERS, reducing lipid accumulation and preventing hepatic steatosis.	[70]
TM	XBP1 (−)	C57BL/6 mice	Exacerbating ERS, inducing hepatocyte death and progression of liver fibrosis, and progressively aggravating liver injury.	[71]
MCD diet	C/EBPβ (−)	C57BL/6 J mice	Alleviating ERS, reducing lipid accumulation, preventing hepatic steatosis, and liver injury.	[117]
TG/TM	c-Jun (−)	Alfp-Cre mice	Exacerbating ERS, inhibiting autophagy, and inducing hepatocyte death.	[118]
TM	LRH1 (−)	Albumin-Cre mice	Exacerbating ERS, causing lipid accumulation and inducing hepatocyte apoptosis.	[119]
KO	ACOX1 (−)	BAC transgenic mice	activating ERS, causing metabolic disorders and inducing cell death, leading to steatohepatitis.	[120]
TM	BAP31 (−)	Mice	Exacerbating ERS, causing lipid accumulation and leading to hepatic steatosis.	[121]
TM	BDNF (−)	Mice	Exacerbating ERS, causing lipid accumulation, inducing cell death, and leading to hepatic steatosis.	[122]
KO	Beclin1 (−)	Zebrafish larvae	activating ERS and inducing hepatocyte apoptosis.	[123]
KO	CBF/NFY (−)	Alb-Cre mice	Activating ERS, cause lipid accumulation, leading to liver cell degeneration and progressive liver injury.	[124]
Hepatectomy	CDK5 (−)	Alb-Cre mice	Exacerbating ERS, causing lipid accumulation, inducing an inflammatory response, resulting in delayed hepatocyte proliferation.	[125]
KO	ERdj4 (−)	C57BL/6 mice	Activate ERS, lead to fetal growth restriction, increased perinatal mortality.	[126]
TM/HFD	GRB10 (−)	Alb-Cre mice	Reduce fatty acid synthesis, reduce hepatic lipid accumulation, prevent hepatic steatosis.	[127]
TM/TG	LCN2 (−)	Primary hepatocytes	Exacerbating ERS, causing lipid accumulation, leading to hepatic steatosis.	[128]
KO	Nrf1 (−)	Mice	Activate ERS, inhibition of proteasome activity, lead to hepatic steatosis.	[129]
HFD	SIRT1 (−)	C57BL/6 J mice	Exacerbating ERS, leading to hepatic steatosis, and obesity.	[130]
HFD/Palmitate	SIRT2 (−)	C57BL/6 mice	Exacerbating ERS, causing lipid deposition, leading to obesity.	[131]
KO	SIDT2 (−)	Mice	Activate ERS, cause lipid deposition, lead to hepatic steatosis.	[132]
Sorafenib	STAT3 (−)	Huh7, Hep3B & C57BL/6 J mice	Exacerbating ERS and inducing cell death.	[133]
HFD	Vinexin β (−)	C57BL/6 mice	Exacerbating ERS, causing lipid accumulation and leading to hepatic steatosis.	[134]

^*^ERS endoplasmic reticulum stress, *HFD* high-fat diet, *KO* knockout, *LPS* lipopolysaccharide, *MCD* *diet* methionine and choline deficient diet, *TG* thapsigargin, *TM* tunicamycin.

### Viral hepatitis

Viral hepatitis is a kind of infectious disease caused by the hepatitis virus. It is considered among the world’s deadliest major infectious disease, along with AIDS, tuberculosis, and malaria [72], seriously threatening human health, especially in underdeveloped countries such as South Asia, Africa, and South America [73]. Hepatitis B virus (HBV) and Hepatitis C virus (HCV) are the main causes of viral hepatitis infection. According to the WHO development plan for eradicating viral hepatitis, the main challenge facing the global response to viral hepatitis is the lack of effective preventive measures and treatment methods [74]. In recent years, many studies have documented that ERS plays an important role in the occurrence and progression of viral hepatitis, which provides novel insights for preventing and treating viral hepatitis.

#### The role of ERS in HBV

HBV is a hepatotropic DNA virus. According to the WHO, as of 2019, about 296 million people worldwide are infected with HBV [72]. Intact HBV particles, also known as Dane particles, with a diameter of 42 nanometers, represent the main carrier of HBV infection and replication. Dane particles are divided into the envelope and the capsid. The envelope is mainly composed of Hepatitis B surface Antigen HBsAg (with three types Large, Middle, and Small), glycoproteins, and lipids. The capsid mainly comprises Hepatitis B core Antigen (HBcAg), Hepatitis B envelope Antigen (HBeAg), DNA polymerase, and double-stranded DNA. HBV gene is about 3.2 kb in length and is part of double-stranded cyclic DNA, with four open reading frames named S, X, C, and P on its DNA strand. These genes encode the viral envelope proteins L-HBsAg, M-HBsAg, S-HBsAg, HBx protein, and polymerase [75].

After infection with HBV, when L-HBsAg is overexpressed, the proportion of three types of HBsAg cannot reach appropriate levels and combine into secretable Dane particles, and the secretion of HBV proteins is blocked and gradually accumulates, inducing ERS. HBsAg can induce apoptosis through the PERK pathway, causing ground-glass degeneration of hepatocytes and hypersensitivity to inflammatory factors, thereby causing liver parenchyma damage [76]. Current evidence suggests that S-HBsAg activates the FGF19-JAK2-STAT3 pathway through the ATF4 pathway, causing the epithelial-mesenchymal transition of hepatocytes, which can promote the occurrence of hepatocellular carcinoma [77]. However, the exact role of the HBx protein remains largely unclear at present, although it is widely thought to be closely related to the infection and replication of HBV. On the one hand, the HBx protein can promote cell survival by inhibiting PERK pathway activation, thereby triggering persistent HBV infection. On the other hand, it can activate cyclooxygenase II, which induces an inflammatory response and aggravates infection symptoms [78]. In addition, Yaming Liu et al. found that HBV or L-HBsAg interacts with apolipoprotein H, which can inhibit the secretion of HBsAg and make it retained in cells, thereby enhancing HBV-induced ERS, which may cause further liver damage and even induce tumors [79].

#### The role of ERS in HCV

HCV is an enveloped positive single-stranded RNA virus of the Flaviviridae family consisting of 9500 to 10,000 bp and does not integrate into the host genome. Interestingly, HCV can encode a protein precursor with a length of 3014 amino acids, which undergoes proteolysis to produce three structural proteins: core protein, envelope protein E1, and E2. The nonstructural proteins of HCV consist of NS2, NS3, NS4A, NS4B, NS5A, and NS5B. Different proteins that compose HCV can regulate the balance between virus infection and cell survival or cell death by activating ERS and its corresponding downstream pathways, leading to adverse outcomes of virus infection, clinical deterioration, and carcinogenesis [80].

It is widely thought that HCV can activate ERS and cause persistent HCV infection, an important process in its progression and carcinogenesis. The core protein of HCV can induce ERS, destroy intracellular Ca^2+^ imbalance and promote the expression of CHOP protein, leading to apoptosis [81]. Importantly, both envelope proteins E1 and E2 of HCV can activate CHOP through the PERK pathway, causing hepatocyte injury. When envelope proteins E1 and E2 accumulate in the ER, GRP78 interacts with them and can transport them into the cytoplasm, which can induce UPR by inducing the synthesis and cleavage of XBP1 [82]. The activation of the UPR by the nonstructural protein NS4B of HCV can transfer ATF6 from ER to the Golgi complex and generate active ATF6 fragments. Moreover, studies have shown that NS4B can induce UPR by inducing the phosphorylation of IRE1α and activating XBP1 [83]. Other studies reported that ERS caused by HCV could impair autophagic flow by inhibiting the fusion of autophagosomes and lysosomes, thereby promoting HCV replication. Although this does not cause apoptosis, what is even more frightening is that the ERS caused by HCV may promote the progression of chronic hepatitis C to liver cancer. Therefore, inhibition of ERS is also considered an important means to treat HCV [84]. Another study reported that HCV core protein and NS5A protein could be degraded by the PERK-eIF2α-ATF4 pathway to counter cellular oxidative stress and apoptosis, which also facilitates HCV replication [85, 86]. In addition, HCV can promote the apoptosis of human embryonic liver stem cells through the eIF2α-CHOP pathway of ERS and promote the transformation of HCV infection to liver cirrhosis and hepatocellular carcinoma [87]. Taken together, the above findings suggest that ERS plays a major role in HCV infection, outcome, and progression.

### Drug-induced liver injury

Drug-induced liver injury (DILI) refers to the direct or indirect damage caused by drugs or their metabolites to the liver. It is one of the major adverse reactions of drugs, and also an important factor limiting the development of new drugs. DILI has been reported frequently, but the most representative ones are still non-specific drugs that need to be taken for a long time, such as efavirenz (an antiviral drug used to treat AIDS), rifampicin (an antimicrobial drug used to treat tuberculosis) and the common and classic antipyretic analgesic acetaminophen.

A growing literature suggests that the non-nucleoside analog reverse transcriptase inhibitor efavirenz can induce significant upregulation of CHOP and GRP78 mRNA and protein levels, phosphorylation of eIF2α, production of XBP1s, and expansion of ER membrane in primary human hepatocytes [88, 89]. Besides, the anti-tuberculosis drug rifampicin can increase the mRNA and protein expression levels of GRP78, PERK, ATF4, and CHOP in LO2 and HepG2, resulting in apoptosis, suggesting that it may induce DILI through ERS pathway [90]. Acetaminophen, a classic antipyretic and analgesic drug, can also induce glutathione depletion in mouse hepatocytes, causing redox imbalance in the lumen, accelerating the phosphorylation of eIF2α, activation of ATF6 and CHOP, and ultimately inducing hepatocyte apoptosis and liver injury by ERS [91]. In addition, Kusama H et al. found that intraperitoneal injection of acetaminophen in mice can cause extensive apoptosis or necrosis of mouse hepatocytes and increased level of ATF6 mRNA, suggesting that acetaminophen-induced hepatotoxicity may also be related to the activation of the ATF6 pathway [92]. In addition to the drugs mentioned above, liver injury caused by traditional herbal medicines remains an important concern. Fructus Cnidium can induce apoptosis by activating ERS and inhibiting the proliferation of LO2 cells [93]. Oxymatrine in *Sophora flavescens* can also induce the occurrence of ERS and the phosphorylation of c-JNK through the excessive accumulation of reactive oxygen species, causing LO2 apoptosis, which can be alleviated by 4-PBA [94]. In vivo studies found that psoralen induces ERS in hepatocytes of female C57 mice, increases liver injury-related factors’ expression, and causes inflammatory infiltration and vacuolar degeneration in hepatocytes [95]. The mechanism of DILI is indeed very complex, involving various mechanisms such as protein dysfunction, inflammation, oxidative stress, mitochondrial damage, and autoimmune response. However, overwhelming evidence suggests that ERS is also an important mechanism causing DILI.

### Liver fibrosis

Liver fibrosis refers to the complex multicellular self-repair process after external stimulation and represents an adverse outcome of various chronic or persistent liver injuries. It often progresses to liver cirrhosis and hepatocellular carcinoma (HCC), which is life-threatening. It is widely believed that the occurrence and development of liver fibrosis are caused by the activation of hepatic stellate cells (HSC) and the excessive deposition of extracellular matrix (ECM). This process is related to the disrupted balance between the production and degradation of ECM proteins [96]. A study found that mice HSC could dysregulate the expression of microRNA 18 A through the PERK pathway of ERS and induce the overexpression of mothers against decapentaplegic homolog 2 (Smad2) to promote liver fibrosis [97, 98]. Transforming growth factor β (TGF-β) is a pro-fibrotic cytokine, considered the core promoter of promoting the occurrence and maintenance of liver fibrosis, and can induce the massive release of ECM and inhibit ECM degradation. TGF-β also plays a key role in HSC activation [99–101]. One study found that TGF-β could activate IRE1α-XBP1 during HSC activation, thereby promoting the development of fibrosis [102]. In addition, adipocytes can cause hepatocyte fibrosis, which is related to the activation of the PERK-eIF2α-ATF4 branch pathway of UPR, and adipocytes promote the progression of fibrosis by enhancing autophagy [103]. Angiotensin II also plays an important role in the progression of liver fibrosis. In this respect, ample evidence corroborates that Aug II can activate the PERK-CHOP pathway in ERS during the induction of liver fibrosis, accompanied by an increase in ECM expression and inflammation [104].

### Liver cancer

Cancer represents a major public health issue worldwide. HCC reportedly has a low survival rate due to the lack of specific drugs and treatments. In this respect, liver cancer was the seventh most common cancer in 2020, with 905,677 new cases, and ranked second for cancer-related deaths (with 830,180 deaths) [105]. Therefore, the quest for more effective ways to treat HCC is of great significance in improving the prognosis of HCC patients. Current studies have found that ERS is closely related to cell cycle regulation, activation and inhibition of proto-oncogenes and tumor suppressor genes, immune response, and changes in the tumor microenvironment during the occurrence and progression of HCC [106–109]. A recent study by Feng et al. suggests that ERS in HCC cells can induce CNYP2, thereby activating three response pathways of UPR, inhibiting the expression of tumor suppressor gene p53 and shortening the cell cycle, ultimately promoting the occurrence of HCC [106]. Another study showed that ERS, when activated in liver cancer tissue, helps HCC cells to deliver exosome microRNA-23a-3p to macrophages, activate the PI3K-AKT pathway by inhibiting phosphatase and tensin homolog, and increase the expression of programmed death ligand 1. Besides, the function of T cells is inhibited, leading to poor immunity and HCC progression [108]. In addition to the occurrence and progression stage, ERS is closely related to invasive HCC. In cancer cells, ERS binds to the promoter by activating PKC and ATF2 to promote the expression of cyclase-associated protein 2 (CAP2). Interestingly, the activation of Rac Family Small GTPase 1 and extracellular regulated protein kinases promotes epithelial-mesenchymal transition (EMT) to enhance the invasive ability of HCC cells [110].

Significant inroads have been achieved in recent years, which have led to new findings on the presence of excessive ERS in NAFLD, viral hepatitis, and DILI. Indeed, persistent ERS leads to the loss of physiological function, cell death of liver cells, and more serious consequences, including liver fibrosis [103]. The activation of HSC and the deposition of ECM are key factors in the process of liver fibrosis, and ERS and UPR are widely involved in these processes [111]. When liver fibrosis progresses further, it eventually progresses to HCC, the outcome of all liver diseases. During HCC, the sustained activation of ERS leads to changes in the expression of proto-oncogenes and tumor suppressor genes, which promotes the progression of liver cancer. In addition, ERS can promote metastasis of liver cancer by activating the expression of related genes. On the other hand, excessive ERS can cause cell death, accounting for ERS-induced liver injury during the early stage of liver injury. However, ERS-induced cell death in HCC can be leveraged to kill cancer cells. Many experiments have shown that ERS permeates the whole process of liver injury. After external stimulation, ERS interferes with lipid metabolism, aggravates virus infection, promotes hepatocyte death, and causes liver injury.

## Therapeutic strategies for ERS in liver disease

It is widely believed that ERS and UPR play a key regulatory role in the occurrence and progression of various liver injuries, suggesting they may be important therapeutic targets for liver injury-related diseases. Indeed, alleviation and activation of ERS may play a role in preventing, diagnosing, and treating liver diseases.

### Alleviation of ERS

ERS plays an important role in the process of liver injury, and when ERS is activated, it can further cause cell death through the UPR, generally considered deleterious in normal tissues, organs, and cells. Researchers have recently attempted to reduce liver damage by inhibiting ERS. For example, 4-PBA, an ERS inhibitor, could alleviate Rifampin-induced cholestatic liver disease by inhibiting ERS and Multidrug resistance-associated protein 2 ubiquitination degradation, thereby reducing serum total bilirubin concentration, suggesting that 4-PBA could be used to treat Rifampin-induced cholestatic liver injury in the treatment of tuberculosis [112]. Besides ERS inhibitors, some traditional herbal medicines have also been found to have the ability to inhibit ERS, thereby exerting hepatoprotective effects. For example, salidroside can inhibit ERS-mediated apoptosis through the IRE1α-JNK pathway, exerting a protective effect against hypoxic liver injury [113].

### Activation of ERS

In addition to inhibiting ERS, the liver can be protected from more serious damage by activating ERS process. Indeed, this strategy may be applied to treating liver cancer. On the one hand, activated ERS can enhance its ability to regulate adverse responses within certain capacities, such as increasing GRP78 protein synthesis to improve its ability to process unfolded proteins or degrading misfolded proteins through RADD and ubiquitination. On the other hand, when ERS is enhanced, the damaged cells can be induced to die to avoid more severe damage. For example, nano-titanium dioxide can induce the apoptosis of liver cancer cells by activating the PERK-ATF6 pathway to treat liver cancer [114]. In addition, Ginsenoside CK, an active ingredient in traditional Chinese medicine Ginseng, can exert its anti-cancer effect by inhibiting the activation of STAT3 and then activating ERS to induce apoptosis of liver cancer cells [115]. Another study found that ERS activation can induce apoptosis of liver cancer cells to exert its anti-cancer effect using proteomics [116].

The accumulation of unfolded or misfolded proteins is generally considered the cause of ERS, which plays an important role in the occurrence and treatment of liver injury and other diseases. However, little is currently known about unfolded or misfolded proteins. Therefore, future studies should target unfolded or misfolded protein accumulation in the ER. In addition, with the development of multidisciplinary cross-integration using genomics, transcriptomic and proteomic technologies, it is feasible to analyze the proteins accumulated in ERS induced by different liver injury diseases to further discover the molecular mechanism of disease occurrence and provide new ideas for precise treatment, which represents a potential new strategy for treating diseases in the future, especially refractory diseases.

## Conclusion and future perspective

**Fig. 5:**
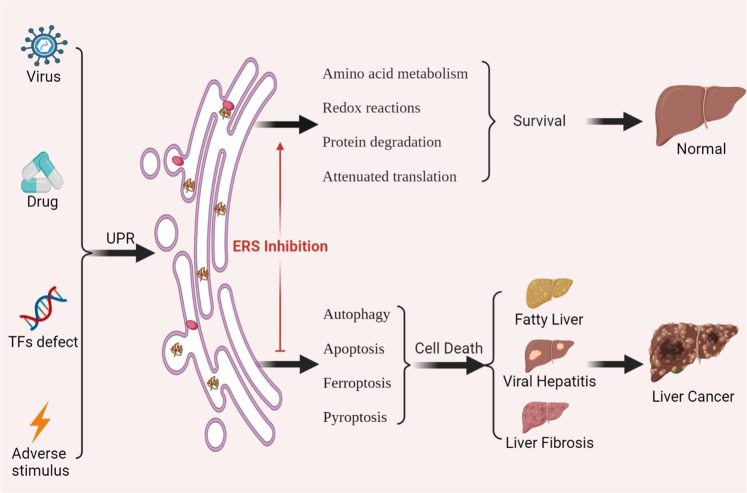
Liver injury and therapeutic pathways involved in UPR. ERS and UPR are commonly induced in the liver in response to stimuli such as viruses, drugs, and high-fat diet (HFD). When stimulation is weak, it can be adaptively regulated by amino acid metabolism, redox reaction, protein degradation, and attenuated translation to maintain the normal physiological function of the liver. When the stimulation is enhanced or prolonged, it will cause cell death and lead to viral hepatitis, fatty liver injury, drug-induced liver injury (DILI), and even hepatocellular carcinoma (HCC). Therefore, ERS can also regulate cell death in the process of liver injury to achieve the therapeutic effect of liver injury.

ERS plays an important role in physiological activities and pathological injury as a highly conserved signal transduction pathway, and has a dual regulatory role in vivo. On the one hand, it can restore ER homeostasis through UPR and promote cell survival. On the other hand, when the external stimuli exceed the regulatory capacity of ER, the downstream UPR signaling is activated to induce cell death (Fig. 5). In recent years, ERS has become a research hotspot. Although significant progress has been achieved in understanding the role of ERS in regulating cell outcomes, body injury, and various diseases, there are still some unanswered questions. How do the three signaling pathways downstream of UPR interact with each other? Is the role of ERS consistent in different stages of liver injury? How does ERS determine its final cell death mode? These issues should be the target of future studies. In addition, treating diseases through the ERS pathway represents a potential new strategy for precision medicine, which could benefit the prevention, diagnosis, and treatment of many intractable diseases.

## Supplementary information

reproducibility checklist

## Data Availability

All data in the current study are available.
